# Anti-Biofilm Activity of a Self-Aggregating Peptide against *Streptococcus mutans*

**DOI:** 10.3389/fmicb.2017.00488

**Published:** 2017-03-24

**Authors:** Juliana M. Ansari, Nabil M. Abraham, Jenna Massaro, Kelsey Murphy, Jillian Smith-Carpenter, Erol Fikrig

**Affiliations:** ^1^Department of Biology, Fairfield University, FairfieldCT, USA; ^2^Department of Internal Medicine, Section of Infectious Diseases, Yale University School of Medicine, New HavenCT, USA; ^3^Howard Hughes Medical Institute, Chevy ChaseMD, USA; ^4^Department of Chemistry and Biochemistry, Fairfield University, FairfieldCT, USA

**Keywords:** biofilm, anti-biofilm, synthetic peptide, *Streptococcus mutans*, Gram-positive cocci, aggregation

## Abstract

*Streptococcus mutans* is the primary agent of dental cavities, in large part due to its ability to adhere to teeth and create a molecular scaffold of glucan polysaccharides on the tooth surface. Disrupting the architecture of *S. mutans* biofilms could help undermine the establishment of biofilm communities that cause cavities and tooth decay. Here we present a synthetic peptide P1, derived from a tick antifreeze protein, which significantly reduces *S. mutans* biofilm formation. Incubating cells with this peptide decreased biofilm biomass by approximately 75% in both a crystal violet microplate assay and an *in vitro* tooth model using saliva-coated hydroxyapatite discs. Bacteria treated with peptide P1 formed irregular biofilms with disconnected aggregates of cells and exopolymeric matrix that readily detached from surfaces. Peptide P1 can bind directly to *S. mutans* cells but does not possess bactericidal activity. Anti-biofilm activity was correlated with peptide aggregation and β-sheet formation in solution, and alternative synthetic peptides of different lengths or charge distribution did not inhibit biofilms. This anti-biofilm peptide interferes with *S. mutans* biofilm formation and architecture, and may have future applications in preventing bacterial buildup on teeth.

## Introduction

The ability of oral streptococci to establish biofilm growth on the surface of teeth is crucial to the formation of dental plaque and dental cavities. *Streptococcus mutans* in particular adheres to teeth and utilizes dietary sucrose to synthesize extracellular insoluble glucans that can serve as scaffolding for other microbes to colonize (Koo et al., 2013). Tooth decay ensues when *S. mutans* and other microbes residing within the plaque biofilm produce acids that erode enamel. Along with the exopolysaccharides glucan and dextran, the composition of *S. mutans* biofilms has been described in detail (Klein et al., 2015), and contains adhesin proteins and extracellular DNA (eDNA) (Liao et al., 2014). Biofilm three-dimensional structure is established when microcolonies of *S. mutans* attached to a substrate multiply and then grow vertically, connecting with one another via the extracellular polymers. Throughout the biofilm matrix, water channels transport nutrients and signaling molecules to the cells (Donlan and Costerton, 2002). This 3D architecture facilitates creation of low-pH microhabitats for oral microbes (Xiao et al., 2012). The presence of and interplay between these molecular polymers, and the water channels, contribute to the stability of the network of the biofilm matrix that supports the cells. Interfering with *S. mutans* biofilm architecture may help prevent formation of oral biofilms that lead to dental caries.

Anti-biofilm molecules have been described with diverse mechanisms of action, such as: inhibiting bacterial adhesion, disrupting intercellular communication, and otherwise dispersing or preventing the establishment of biofilms (Bjarnsholt et al., 2013; Rendueles et al., 2013; de la Fuente-Núñez et al., 2014). In contrast to antimicrobial peptides, which are important tools to combat oral pathogens (Chen et al., 2014; Ding et al., 2014), some anti-biofilm agents do not directly kill bacteria, but instead interfere with their biofilm-forming capacity. Without the protective assemblage of the biofilm matrix, bacterial cells are more readily accessible and susceptible to antibiotics or disinfectants (Basak et al., 2013; Otter et al., 2015). Since early biofilm establishment by oral streptococci often precedes the attachment of other oral pathogens, inhibiting these biofilms may help prevent further disease (Xiao and Koo, 2010). Anti-biofilm molecules with activity against *S. mutans* and other oral streptococci include natural products such as alpha-mangostin (Thi et al., 2014), salivary mucin glycoprotein (Frenkel and Ribbeck, 2015), polysaccharide-degrading enzymes (Otsuka et al., 2015), MPC-polymers (Hirota et al., 2011), and small molecules that antagonize glucosyltransferases (Chen et al., 2015).

Synthetic peptides represent a promising class of anti-biofilm molecules, with activity against a variety of pathogens (Di Luca et al., 2015; Cardoso et al., 2016; de la Fuente-Núñez et al., 2016; Pletzer and Hancock, 2016). A cationic synthetic peptide, called P1, derived from the *Ixodes* tick antifreeze glycoprotein (IAFGP), demonstrates potent anti-biofilm activity against *Staphylococcus aureus*, and confers an antivirulence effect in a catheter infection mouse model (Heisig et al., 2014). Given its strong effect against *S. aureus*, we investigated the ability of peptide P1 to combat *Streptococcus mutans* biofilm formation. Antivirulence peptide P1 (distinct from the streptococcal adhesin protein that is called P1 or AgI/PAc) is a 24-amino acid synthetic peptide designed with repeated motifs found in the full IAFGP antifreeze glycoprotein sequence (Heisig et al., 2014) (Supplementary Table 2). Its sequence contains a positively charged N-terminal “PARKAR” motif followed by six “Ala-Ala-Thr” (AAT) repeats, a tripeptide repeat that is also characteristic of fish antifreeze glycoproteins (Neelakanta et al., 2010; Nagel et al., 2012). Biofilm assays were conducted *in vitro* in tissue culture plates and on glass coverslips, and *S. mutans* biofilms were also tested on saliva-coated hydroxyapatite (sHA) discs, which mimic the tooth surface. Here we demonstrate that peptide P1 disturbs the biofilm architecture in *S. mutans*, resulting in a drastic reduction of attached biofilm biomass.

## Materials and Methods

### Peptide Synthesis and Bacterial Cultures

Peptides were synthesized by Genscript (Piscataway Township, NJ, USA) at >80% purity. Peptides P1 and sP1 matched previously published amino acid sequences (Heisig et al., 2014) and the alternative peptide sequences P0, P1–4, P1–7, and P17 were designed as variations of the P1 sequence (**Figure 6** and Supplementary Table 2). Lyophilized peptides were diluted in phosphate-buffered saline (PBS) to a stock concentration of 1 mg/ml and used immediately or frozen at -20°C in 100 μl aliquots. Streptococci bacteria (*Streptococcus mutans* ATCC 25175, *Streptococcus oralis* ATCC 9811, and *Streptococcus salivarius* ATCC 13419), Gram-negative bacteria (*Alcaligenes faecalis* ATCC 8750, *Enterobacter cloacae* ATCC 23355, and *Salmonella typhimurium* ATCC 29629), and Gram-positive bacterial strains (Abraham et al., 2017) were obtained from Yale University or Presque Isle Cultures (Erie, PA, USA). Bacterial cultures were maintained on Brain Heart Infusion (BHI) agar or broth (for streptococci), and Tryptic Soy agar or broth (TSA, TSB).

### Biofilm Microplate Assay and Viability Assay

Biofilm assays were performed as previously described (Merritt et al., 2011). Overnight cultures of *S. mutans* grown in BHI medium added to BHI+1% sucrose (BHI-S) at a concentration of 3–5 × 10^6^ cfu/ml [by either a 1:100 dilution or normalizing to an optical density at 600 nm (OD_600_) of 0.015]. The bacterial suspension was inoculated into quadruplicate wells of a 96-well tissue culture plate and P1 peptide or control sP1 peptide was added at a final concentration of 0.1 mg/ml (46 μM). Bacteria were incubated overnight at 37°C with a CO_2_ GasPak (BD, Franklin Lakes, NJ, USA). Crystal violet staining of biofilm plates was performed using standard protocols (Merritt et al., 2011), as follows: Non-adherent cells were washed off by submerging the plate in water, and adherent cells were stained with 0.1% crystal violet, washed twice with water, dried, and resuspended in 33% acetic acid for 10 min. The absorbance of the resuspended solution was measured at 590 nm on a BioTek microplate reader. For the other Gram-negative and Gram-positive bacterial strains, the assay was identical except that the 96-well plates were incubated aerobically, and the biofilm culture media was TSB + 1% glucose for Gram-negatives, and BHI+1% glucose for Gram-positives.

To assess the bactericidal activity of P1 against *S. mutans*, overnight cultures of *S. mutans* were inoculated into 12-well or 24-well plates at a 1:100 dilution in BHI-S containing 0.1 mg/ml of P1 peptide, control sP1 peptide, or no peptide, and incubated overnight at 37°C with a CO_2_ GasPak. The *S. mutans* cells were collected in two ways: first, as a total (combined) sample, pooling all cells in both the supernatant (planktonic) and biofilm fraction; secondly, the supernatant (non-adherent) and biofilm fractions were separated and processed separately. For the combined sample, the attached biofilm cells were scraped off the bottom using a sterile 1.8 cm cell scraper, resuspended, and the entire contents of the well were transferred to a microcentrifuge tube. An additional 500 μl of PBS was added to the well and re-scraped to pick up any remaining adherent cells. For the separated fractions, the supernatant (containing planktonic bacteria) was pipetted off and transferred to a 1.5 ml tube, then 500 μl of PBS was added gently to the well to rinse off non-adherent cells, and transferred to the same “supernatant” tube. The biofilm cells were resuspended into 1 ml of PBS using a cell scraper, transferred to a new tube, and an additional 500 μl of PBS was added to the well and re-scraped to collect any remaining adherent cells. All samples (total, supernatant, or biofilm) resuspended in a total volume of 1.5 ml each, were briefly sonicated to break up cell clumps (10 pulses at 30–40% duty cycle), then serially diluted in PBS to 10^-7^, spread-plated on BHI agar, and incubated for 48–72 h at 37°C with a CO_2_ GasPak. After counting colonies, the number of colony-forming units (cfu/ml) was calculated for each sample.

### Saliva-Coated Hydroxyapatite (sHA) Disc Experiments

The sHA disc procedure was carried out according to published protocols (Lemos et al., 2010). We prepared authors’ own clarified saliva for each experiment as follows: ~10 mL of saliva was collected in a Falcon tube, stored on ice, and mixed with an equal volume of Adsorption Buffer (AB; 50 mM KCl, 1 mM potassium phosphate, 1 mM CaCl_2_, 0.1 mM MgCl_2_, pH 6.5), followed by the addition of 10 μl of 0.1 M phenylmethyl-sulfonyl fluoride (PMSF). The mixture was centrifuged at 5500 × *g* for 10 min at 4°C. The supernatant (containing clarified whole saliva) was pipetted off carefully and filtered through a 0.22 μm PES low protein-binding filter (Steriflip, EMD Millipore, Billerica, MA, USA) and used immediately or stored at 4°C. Sterile 5-mm diameter hydroxyapatite (HA) discs (3D Biotek, Bridgewater, NJ, USA) were aseptically placed into a sterile 15 ml tube containing 5–10 mL of clarified saliva and rotated at 37°C for 1 h to coat discs with saliva. The sHA discs were then placed vertically in custom-made wire holders in pre-seeded wells of the 96-well culture plates. Triplicate wells were inoculated with a final volume of 200 μl of *Streptococcus mutans* in BHI-S (diluted to an OD 600 nm of 0.015), with either P1 or control sP1 peptides at 0.1 mg/ml. The plate was incubated at 37°C with a CO_2_ GasPak for 24 h. Discs were removed with sterile forceps, dip-washed three times in 0.89% NaCl, and placed in glass tubes containing 1 mL of 0.89% NaCl. The glass tubes were distributed in a custom Styrofoam rack and subjected to a 10-min ultrasonic bath. Following the ultrasonic bath, the detached cell suspension was transferred into microfuge tubes and sonicated (2x 5 pulses, on ice) to break up clumps of cells. Cells were serially diluted in PBS to 10^-5^, then plated on BHI agar and incubated at 37°C with a CO_2_ GasPak. After 48–72 h of incubation, *S. mutans* colonies were counted and cfu/disc was calculated.

### Peptide Binding Assay and Immunoblot Analysis

Approximately 1.5 × 10^7^ cells of *S. mutans*, from an overnight culture growing in BHI, were pelleted and washed in PBS and resuspended in 100 μL PBS. His-tagged P1 or sP1 peptides (GenScript, Piscataway Township, NJ, USA) were added to the cultures at 0.1 mg/mL. Bacteria-peptide mixtures were incubated for 10 min at 37°C. Cells were pelleted by centrifugation, the supernatant (unbound fraction) was removed, and the pellet-associated (bound) fraction was washed with 0.1% Triton X-100 (Sigma–Aldrich, St. Louis, MO, USA) in PBS. The pellet (Associated) fraction and the supernatant (unbound) fraction were spotted directly onto a nitrocellulose membrane for immunoblot analysis. Bacterial bound (Associated) or unbound (Supernatant) peptide was detected using a monoclonal His-tagged antibody (Clontech, Mountain View, CA, USA). Cell wall bound fraction (Associated) versus unbound (Supernatant) bacterial fractions were distinguished using a polyclonal anti-Wheat Germ Agglutinin (WGA) (Vector Laboratories, Burlingame, CA, USA) antibody that detects bacterial peptidoglycan. Primary antibodies for both His and WGA were used at a 1:2000 dilution. Appropriate IR-conjugated LICOR secondary antibodies were used at a 1:5000 dilution and detected using the LI-COR Odyssey system (LI-COR, Lincoln, NE, USA). To normalize for any differences in reactivity between P1-His and sP1-His with the anti-His antibody, we quantified the bound signal intensity (Associated) and represented it as a percentage of the total signal (Associated + Supernatant):

$$\mathrm{Relative Signal Intensity}=\frac{\left[\mathrm{Associated}(\mathrm{P1 or P1})\;\mathrm{Signal}\right]}{[\mathrm{Associated}+\mathrm{Supernatural signal}]*100}.$$

### Phase Contrast and Fluorescent Microscopy

Overnight cultures of *S. mutans* were inoculated at a 1:100 dilution in BHI-S with 0.1 mg/ml of P1 peptide or control sP1 peptide. Biofilms were formed either on coverslips in 6-well plates, or directly on the surface of 12-well or 24-well tissue culture plates (Corning, Corning, NY, USA). Bacteria were incubated overnight at 37°C in containers with a CO_2_ GasPak. Biofilm cultures and peptides in solution in tissue culture plates were viewed with phase contrast microscopy using a Zeiss Axio Vert.A1 microscope. For eDNA staining, a Miami Orange DNA-binding fluorescent probe (Kerafast, Boston, MA, USA) was used at a 1:200 dilution in the well, and viewed with a TRITC fluorescent filter. Images were captured using either a mounted camera with SPOT imaging software (SPOT Imaging, Sterling Heights, MI, USA) or an iPhone camera (Apple, Cupertino, CA, USA) mounted to the eyepiece of the microscope with a MiPlatform (Scientific Device Laboratory). Scale bars were calibrated using a stage micrometer.

### Scanning Electron Microscopy (SEM)

*S. mutans* was inoculated at a 1:100 dilution into 500 μl of BHI-S in a 12-well tissue culture plate, with glass coverslips or silicon wafers aseptically placed at the bottom to allow for biofilm formation. Peptide P1 at 0.1 mg/ml was added to half of the samples, and the other half contained 0.1 mg/ml control peptide sP1. After 24 h of incubation with CO_2_ at 37°C, the coverslips and wafers were gently rinsed twice in 0.12 M phosphate buffer for 10 min. Coverslips/wafers were suspended for 1 h in a fixative solution containing 1.5% glutaraldehyde and 1% formaldehyde in 0.12 M phosphate buffer, followed by two rinses in 0.12 M phosphate buffer. Coverslips and wafers were then fixed in 1% OsO_4_ in 0.12 M Phosphate for 30 min, followed by a di H_2_O rinse. Next, the coverslips/wafers went through an ethanol dehydration process, suspended in the following concentrations of ethanol for 20 min each: 30, 50, 70, 95, 100% ethanol. Following the 100% ethanol, samples were moved to the critical point dryer in liquid CO_2_. After critical point drying, the coverslips/wafers were sputter coated with gold palladium in argon gas. Biofilm samples were imaged at the UConn BEML facility using an FEI Nova NanoSEM 450. Images were photographed at magnifications ranging from 500× to 50,000×, and the 3084× magnification was selected for bacterial chain length measurement. Twenty additional images from the P1 and control sP1 biofilms were taken at 3084×, and two representative images per treatment were used to estimate streptococcal chain length. Chains of cells with a clear beginning and end (30 chains per treatment group) were randomly selected and chain length was measured with ImageJ software (Schneider et al., 2012).

### Fourier Transform Infrared (FTIR) Spectroscopy

Synthetic peptides P1, P17, and sP1 were diluted to a concentration of 1 and 0.1 mg/ml in PBS. Aliquots of 8 μl per sample were dried as thin films directly on the ATR surface of a Bruker Alpha Platinum-ATR FTIR (Bruker Daltonics, Billerica, MA, USA). To scan for absorption peaks indicating the presence of β-sheets, spectra were recorded in the range of 1800–1500 cm^-1^ at a resolution of 2 cm^-1^, with 50 scans per sample.

### Statistical Analysis

Experiments were repeated independently at least three times, and one representative experiment or pooled data are shown. Data were analyzed using GraphPad Prism version 6.0 software (GraphPad Software, Inc., La Jolla, CA, USA). Statistical significance was determined using Students *t*-test with Welch’s correction (when comparing two groups) or One-Way ANOVA with Dunnett’s or Sidak’s multiple comparison post-test.

## Results

### Antivirulence Peptide P1 Reduces *S. mutans* Biofilm in Microplates and on sHA Discs

**FIGURE 1:**
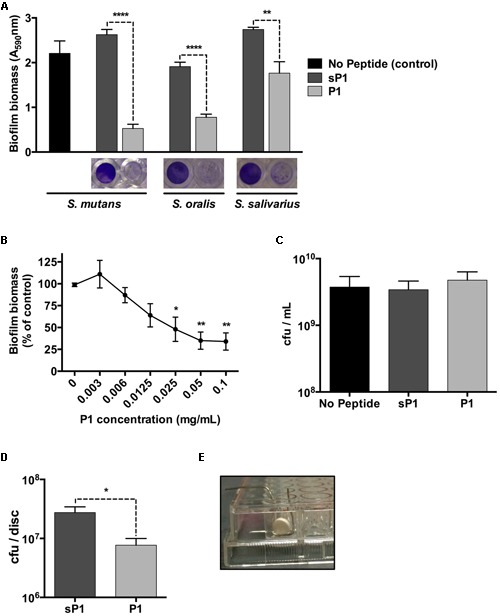
**Anti-biofilm effect of peptide P1 in microplate assay and on sHA discs. (A)** Peptide P1 reduces biofilm formation by *Streptococcus mutans* and oral streptococci in microplate assay. Cultures were incubated with peptide P1 and control peptide sP1 at 0.1 mg/ml final concentration. Biofilm biomass in microplate wells was stained with crystal violet (images below graph). Results are combined from three replicate experiments, and error bars indicate standard error of the mean. **p < 0.01, ****p < 0.0001, Student’s *t*-test. **(B)** Determination of minimum biofilm inhibitory concentration of peptide P1 against *S. mutans*. Biofilm inhibitory activity of P1 was measured by testing decreasing peptide concentrations in a microplate assay. Results were combined from three experiments, with biofilm biomass normalized to a percentage of the control. **(C)** Viability of *S. mutans* in the presence of peptide P1. Total *S. mutans* biomass (combined planktonic and biofilm cells from well) of cultures grown overnight in Brain Heart Infusion + 1% sucrose (BHI-S) without peptide or with 0.1 mg/ml P1 or sP1 was collected, diluted and plated, and cfu/ml was quantified for each condition. **(D)** Peptide P1 reduces *Streptococcus mutans* biofilm formation on saliva-coated hydroxyapatite (sHA) discs. Biofilm cfu/sHA disc showing combined results from three replicate experiments (total *n* = 9 discs/treatment). Peptide P1 and control peptide sP1 were used at 0.1 mg/ml final concentration. Error bars show standard error of the mean. *p < 0.05, using Student’s *t*-test. **(E)** Custom-made wire holder holding the hydroxyapatite disc vertically in the well of a 96-well plate.

The biofilm microplate assay using crystal violet staining is a rapid and convenient test for static biofilm formation. We cultured *Streptococcus mutans* cultures in BHI medium containing 1% sucrose, along with peptide P1 or a randomized scrambled peptide, sP1, a previously published negative control (Heisig et al., 2014). Peptides were used at a final concentration of 0.1 mg/ml (46 μM). As shown in **Figure 1**, when co-incubated with peptide P1 overnight, the biofilm biomass of *Streptococcus mutans* was reduced by approximately 80% compared to the control biofilms (sP1 peptide or no peptide) (**Figure 1A**). Likewise, incubation with P1 under the same conditions also reduced biofilm formation by two other oral streptococci, *Streptococcus oralis* and *Streptococcus salivarius* (**Figure 1A**). To determine the minimum effective concentration for biofilm inhibition of *S. mutans*, we tested decreasing concentrations of peptide P1. Significant anti-biofilm activity was observed at concentrations down to 25 μg/ml (**Figure 1B**), however, 0.1 mg/ml gave consistent results and was used for all other experiments. Despite inhibiting biofilm formation, peptide P1 was not bactericidal against *S. mutans*, similar to the effect on *S. aureus* (Heisig et al., 2014). Viability assays revealed a similar number of total colony-forming units present in control cultures and cultures incubated with P1, after dilution and plating of the entire contents of the tissue culture well (biofilm and non-adherent cells) (**Figure 1C**). As expected, when cells from the biofilm and non-adherent (supernatant) fractions were processed and quantified separately, there were ~80% fewer cfu/ml in the biofilm-associated fraction of the P1-incubated *S. mutans* cultures compared to controls, and more cfu/ml in the non-adherent (supernatant) fraction (Supplementary Figure 1).

To investigate the effect of peptide P1 on *S. mutans* biofilms in an *in vitro* tooth model, we prepared sHA discs, an established model that mimics tooth enamel coated with a salivary pellicle (Koo et al., 2010). The sHA discs were coated with clarified saliva using a standard method (Lemos et al., 2010) and incubated vertically for 24 h in handmade wire holders in 96-well plates (**Figure 1E**) with *S. mutans* in BHI-S with peptide P1 or sP1. After incubation, attached biofilm bacteria were collected from the discs (by washing to remove loosely attached bacteria, then placed in tubes in an ultrasonic bath to detach biofilm bacteria from the discs). The detached biofilm bacterial suspension was sonicated to break up clumps of cells before plating onto BHI agar. When *S. mutans* was cultivated on the discs in the presence of P1, we observed a 70% decrease in the number of biofilm colony-forming units (cfu) per disc (7.7 × 10^6^ cfu/disc) compared to the sP1 control (2.8 × 10^7^ cfu/disc) (**Figure 1D**). We did not observe a significant difference in 48-h biofilms of *S. mutans* (data not shown).

### Peptide Binding to *S. mutans* and Biofilm Inhibition of Other Bacteria

**FIGURE 2:**
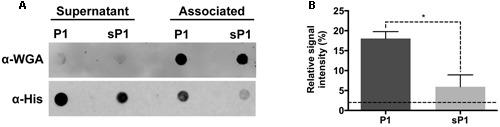
**Immunoblot assay of *S. mutans* binding to His-P1 peptide or His-sP1 control peptide. (A)** Peptides were detected using a monoclonal His-tagged antibody (α-His). Cell wall bound fraction (Associated) versus unbound (Supernatant) bacterial fractions were distinguished by using a polyclonal anti-Wheat Germ Agglutinin antibody (α-WGA). **(B)** Quantification of bound antiserum of His-tagged peptide in the cell-associated fraction. The Relative Signal Intensity is the signal of “associated” fraction as a percentage of total signal detected for each peptide [associated/(supernatant + associated)]*(100). Lower dotted line indicates the limit of detection by the LICOR Odyssey system. *p < 0.05, using Student’s *t*-test.

The full-length IAFGP protein, from which P1 was designed, was previously shown to bind to the cell surfaces of a range of bacteria, including both Gram-positive and Gram-negative species (Heisig et al., 2014). Direct binding between both IAFGP and peptide P1 and cell wall peptidoglycan of *S. aureus* and *Enterococcus faecalis* was recently discovered (Abraham et al., 2017). To establish whether peptide P1 can bind to *S. mutans* cells, we performed an immunoblot assay with His-tagged peptide P1 or sP1. The His-tagged peptides were incubated with a culture of *S. mutans*, then the cells were spun down, washed, and the cell-associated and unbound fractions were probed with anti-His antibody. As shown in **Figure 2**, there was strong binding detected between His-P1 peptide with the cell-associated *S. mutans* fraction (anti-His, “Associated”), while only a trace amount of His-sP1 control peptide was detected bound to cells.

**FIGURE 3:**
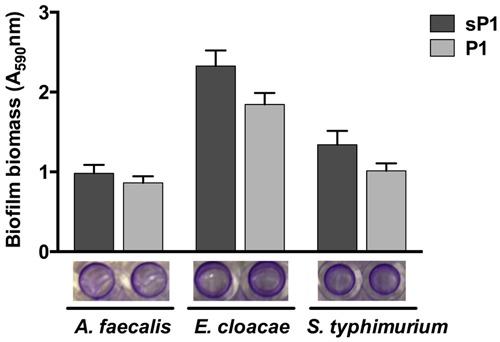
**Peptide P1 activity against biofilms of Gram-negative bacteria.** Biofilm biomass in microplate wells was stained with crystal violet (images below graph). Results are combined from three replicate experiments with cultures grown in TSB + 1% glucose, using peptides P1 and sP1 at 0.1 mg/ml final concentration. Error bars indicate standard error of the mean.

Since P1 peptide binds to *S. mutans*, and IAFGP binds to a range of bacteria (Heisig et al., 2014), we wished to investigate the anti-biofilm effect of P1 against other bacteria. Recently, Abraham et al. (2017) reported that P1 had strong anti-biofilm activity against biofilms of Gram-positive species, and no effect on *Escherichia coli* and *P. aeruginosa* biofilm formation (Abraham et al., 2017). We tested the peptide against three additional biofilm-forming Gram-negative strains, *A. faecalis, Enterobacter cloacae*, and *Salmonella typhimurium*, and did not detect any statistically significant biofilm inhibition (**Figure 3**). Supplementary Table 1 summarizes the findings from both studies on peptide P1 against the Gram-positive and Gram-negative bacteria tested thus far. Biofilms of Gram-positive species were reduced by 35–80%, and statistically significant reduction of biofilm by peptide P1 could be detected at concentrations as low as 5 μg/ml against *S. oralis*, and 10 μg/ml against *S. aureus* (Supplementary Table 1).

### P1-Treated Biofilms have Disconnected Exopolymeric Matrix and Shorter Chains of Streptococci.

**FIGURE 4:**
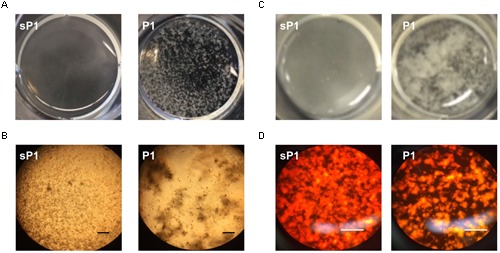
**Clumping and detachment of exopolymeric matrix in the presence of P1. (A)** *S. mutans* cultures in well of 12-well plate after overnight incubation with peptide P1 or sP1 at 0.1 mg/ml. Detached chunks of floating matrix material are visible in P1-treated sample (no magnification). **(B)** Cells and exopolymeric matrix (same samples from **A**) viewed through 10× objective with phase contrast microscopy. Bar = 100 μm. Cells and matrix appear dark, clear areas show lack of biofilm attached to well. **(C)** Matrix clumping in wells of 24-well plate. **(D)** Biofilm eDNA (same samples from **C**) stained with fluorescent Miami Orange probe and viewed under fluorescent light with 20× objective. Bar = 100 μm.

When *S. mutans* biofilms were allowed to form in the wells of 12- or 24-well tissue culture plates, cultures in the presence of peptide P1 exhibited large aggregates of cells and exopolymeric matrix, that detached readily from the surface (**Figure 4** and Supplementary Figure 1). Control sP1-treated biofilms were more uniform and firmly attached, with a continuous network of cells and exopolymeric matrix material (**Figure 4** and Supplementary Figure 1). To visualize eDNA in the biofilm matrix, we stained the total contents of the wells (adherent and suspended) with a fluorescent DNA-binding dye, and observed the same pattern of unevenly clumped matrix material with less attached to the surface of the well (**Figure 4D**).

**FIGURE 5:**
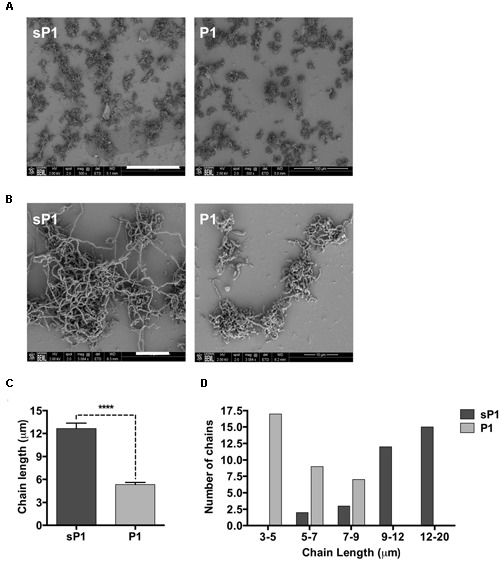
**Scanning electron microscopy of *S. mutans* biofilm. (A)** Reduced adherent material in biofilms formed in the presence of 0.1 mg/ml peptide P1; final magnification 500×, bar = 100 μm. **(B)** Biofilms in P1-treated sample display shorter streptococcal chain lengths; final magnification 3084×, bar = 10 μm. **(C)** Average streptococcal chain length under each treatment, measured with ImageJ software. ****p < 0.0001, Student’s *t*-test. **(D)** Distribution of streptococcal chain lengths under each treatment.

To further examine the altered biofilm structure of *S. mutans* treated with peptide P1, we performed SEM to look for differences in biofilm architecture and cell morphology at high resolution. At 500× magnification, there were fewer clumps of attached *S. mutans* microcolonies in the P1-treated sample (**Figure 5A**). At 3084× magnification, striking differences in streptococcal chain length were apparent between the samples, with the control cells forming significantly longer chains that were extensively interlinked (**Figure 5B**). We measured chain lengths using ImageJ software and found that the majority of streptococcal chains in the P1-incubated biofilm were less than 6 μm long, while chains of cells in the control group were more than twice as long, with most measured at 10–16 μm (**Figures 5C,D**). The representative images in **Figure 5B** illustrate that cells of control biofilms form a more interconnected network, while cells incubated with P1 exist in discontinuous clumps.

### Anti-Biofilm Activity Correlates with Peptide Sequence and Aggregation in Solution

**FIGURE 6:**
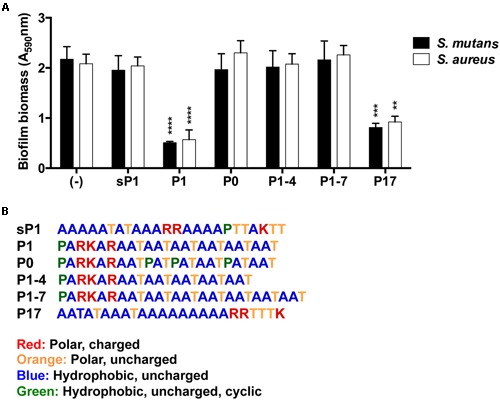
**Effect of alternative peptide sequences on biofilm formation. (A)** Biofilm biomass of *S. mutans* and *S. aureus*, with results combined from three replicate experiments. All peptides were used at 0.1 mg/ml final concentration. Error bars indicate standard error of the mean. **p < 0.01, ***p < 0.001, ****p < 0.0001. **(B)** Amino acid sequences of synthetic peptides with color-coded amino acid properties.

To explore whether altering the sequence of P1 could enhance or eliminate its anti-biofilm activity, we designed modified peptide sequences and tested their anti-biofilm effect on *S. mutans* and *S. aureus*. **Figure 6** lists the alternative peptide sequences with color-coded amino acid residues. Additional details including peptide molecular weights and net charges can be found in Supplementary Table 2. Peptide P0 directly matches the repeated sequence in the full length IAFGP protein (with ‘PATAAT’ instead of ‘AATAAT’). Peptides P1–4 and P1–7 have fewer (4) or more (7) ‘AAT’ repeats, respectively, than P1. P17 is a different randomized scrambled peptide that lacks the proline residue. As seen in **Figure 6**, each peptide had similar effects against both *S. mutans* and *S. aureus* at 0.1 mg/ml in the microplate crystal violet assay. None of the peptides with alterations in the ‘AAT’ repeats (P0, P1–4, or P1–7) reduced biofilm formation. The only variant peptide that showed some anti-biofilm activity was P17, which is a scrambled version of P1 that lacks the proline residue, and contains the three positively charged amino acids (R,R,K) at the C-terminus. Its effect was slightly less potent than that of P1.

**FIGURE 7:**
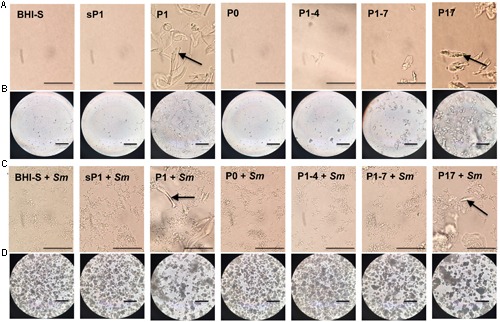
**Aggregation of peptides in solution.** Images were taken with phase contrast microscopy through the bottom of a 24-well tissue culture plate. All peptides were added at 0.1 mg/ml final concentration in BHI+1% sucrose (BHI-S). **(A)** Peptides in solution after 6 h incubation; bar = 50 μm. **(B)** Peptides in solution after 24 h incubation; bar = 100 μm. **(C)** Peptides in solution with *S. mutans* biofilm after 6 h incubation; bar = 50 μm. **(D)** Peptides in solution with *S. mutans* biofilm after 24 h incubation; bar = 100 μm. Arrow = visible aggregated peptide.

In addition to sharing the 24-aa length and +3 net charge at the terminus, P1 and P17 displayed another common property. These peptides form visible aggregates in BHI-S solution, even without bacteria present. **Figure 7** shows microscopic images of each peptide in solution, with and without *S. mutans* bacteria, at 6 and 24 h of incubation. **Figures 7A,C** display the conspicuous self-aggregation of peptides P1 and P17 (arrows). After the bacterial biofilm forms, the wells treated with P1 and P17 display floating chunks of matrix material that appear to associate directly with the aggregated peptide.

**FIGURE 8:**
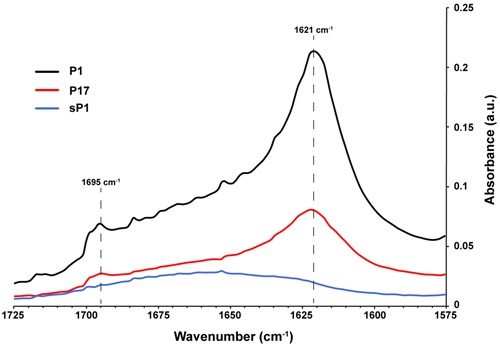
**Secondary structure of peptides in solution using FTIR.** Fourier transform infrared spectroscopy (FTIR) spectra of peptides P1, P17, and negative control sP1 at 1 mg/ml, with peaks at 1621 cm^-1^ indicative of β-sheet formation.

The ability to self-aggregate indicates that P1 and P17 may have different secondary and tertiary structure than the other peptides, depending on the relative locations of proline and the charged and uncharged amino acids in each sequence (**Figure 6**). We used Fourier transform infrared spectroscopy (FTIR) to assess the secondary structure of these two peptides compared to the negative control, sP1 (Haris and Chapman, 1995). The assembly of P1 and P17 peptide monomers into higher order β-sheet structures were confirmed by the presence of a peaks at 1621 and 1695 cm^-1^ (**Figure 8**), characteristic for antiparallel β-sheet formation (Cerf et al., 2009).

## Discussion

The synthetic peptide P1, designed from a tick antivirulence protein, strongly reduced biofilm formation by the oral pathogen *Streptococcus mutans* in an *in vitro* microplate assay and the sHA disc tooth model. Biofilm structure was drastically disrupted in *S. mutans*, with P1-incubated biofilms displaying sparse, irregular attachment, and chunks of matrix and cells that detached into the culture medium. In addition, the chains of streptococcal cells remaining attached in P1-treated biofilms were shorter and less interconnected than control cell chains, suggestive of a disturbance in the 3D structure of the biofilm. Peptide P1 formed aggregates in solution, and alternative peptides containing variations of the repeated ‘AAT’ sequences did not possess anti-biofilm activity, with the exception of P17, a peptide that also aggregated and formed similar secondary structure to P1.

Combining this evidence with the direct binding observed between P1 and *S. mutans* cells, we propose a model in which peptide P1 binds to the bacterial surface and interferes with the proper secretion and/or intermolecular interaction of key extracellular polymers in the biofilm matrix, preventing strong attachment to the surface and connection between microcolonies in the biofilm (Koo et al., 2010). In biofilms of Gram-positive bacteria, interactions between exopolysaccharide (acidic, basic, or neutral), proteins, and the negatively charged eDNA or teichoic acids dictate the stability of the exopolymeric matrix (Otto, 2014). In *S. mutans*, eDNA plays an important role in maintaining the mechanical stability of biofilms (Liao et al., 2014), and interactions between eDNA in the biofilm matrix and the cation calcium (Ca^2+^) were shown to enhance bacterial aggregation and biofilm stability in several species (Das et al., 2014). As a positively charged peptide, P1 may affect interactions between the eDNA and exopolysaccharides, perturbing the typical bonding by altering the net charge at the surface of cells. Disrupted stability is evidenced by the floating chunks of cells and matrix that are readily washed away, rather than firmly attached to the substrate as intact biofilm.

The binding of peptide P1 at the cell surface may also interfere with the secretion and cell wall attachment of components of the biofilm exopolymeric matrix. Antivirulence P1 peptide has recently been shown to bind to the D-ala residue of the pentapeptide of *S. aureus* peptidoglycan (Abraham et al., 2017). Since *S. mutans* shares the same D-ala residue in its peptidoglycan pentapeptide, this could potentially be the location of binding between P1 and the cell surface of *S. mutans.* Microscopic evidence (fragmented matrix and shorter chain length) suggests possible defects in cell envelope processing, which could be a result of P1 altering the activity of peptidoglycan-binding enzymes such as autolysin or sortase. The sortase enzyme SrtA, which covalently links LPxTG motif-containing proteins to the cell wall, is critical for biofilm formation in *S. mutans* (Lévesque et al., 2005). SrtA governs the appropriate cell surface localization of biofilm adhesion proteins (Oli et al., 2012), and plays a role in regulating eDNA in *S. mutans* biofilms (Liao et al., 2014). Glucosyltransferase enzymes secreted by *S. mutans* are important for early attachment and glucan synthesis (Koo et al., 2010), and their activity might be impacted by cell surface-bound peptide P1. The mode of anti-biofilm activity by P1, with effects against various Gram-positive biofilms (Supplementary Table 1), likely differs depending on the composition of the exopolymeric matrix and the key steps in biofilm maturation. However, the absence of P1 anti-biofilm activity against Gram-negative bacteria observed in this study (**Figure 5**), and by Abraham et al. (2017) against *E. coli* and *P. aeruginosa* (Supplementary Table 1) implies that P1 may interfere with conserved elements of the peptidoglycan, protein secretion and/or cell surface remodeling processes shared by certain lineages of Gram-positive bacteria.

We propose that the peptide’s secondary structure and self-aggregation are critical to its activity, because the anti-biofilm effect was lost with shorter or longer versions of P1 (peptides P1–4 and P1–7), or peptides lacking a positive net charge at one terminus (peptides sP1 and P0) (**Figure 6** and Supplementary Table 2). The strongest anti-biofilm effect was correlated with visible peptide aggregation (**Figure 7**) and formation of antiparallel β-sheets (**Figure 8**). Antiparallel β-sheets contribute to the quaternary structure of amyloid fibrils (Cerf et al., 2009), and functional amyloid proteins are produced by many bacteria to perform different cellular functions (Blanco et al., 2012). Amyloid structures are now recognized for playing key roles in stabilizing the exopolysaccharide matrix of biofilms of bacteria (Taglialegna et al., 2016a), including *S. mutans* (Oli et al., 2012) and *S. aureus* (Taglialegna et al., 2016b). In *S. mutans*, a major constituent of the *S. mutans* biofilm matrix was shown to be an amyloid-forming protein (Oli et al., 2012). This Ala-rich protein, known as “antigen I/II, PAc, or P1” (not to be confused with the synthetic peptide P1 used in this study), along with other cell-surface-localized amyloidogenic proteins, are important for biofilm stability (Oli et al., 2012; Besingi et al., 2017). The Ala-rich, β-sheet forming properties of the synthetic P1 peptide may interfere with native amyloid-forming proteins. Aggregation and cell surface binding by P1 impede critical intermolecular interactions needed for proper *S. mutans* biofilm architecture, negatively impacting biofilm stability. *In silico* analyses of antimicrobial peptide sequences suggest that aggregation-prone regions are hotspots for the evolution of antimicrobial activity, when cationization in these regions results in amphipathic helical peptides (Torrent et al., 2011). A compendium of biofilm-active antimicrobial peptides, with their effects on medically relevant species, can be found in the open-access “BaAMPs” database (Di Luca et al., 2015). Amino acid composition analysis of peptides from this database revealed that biofilm inhibitory peptides preferentially contained positively charged residues (Arg and Lys) and that dipeptides such as “RK” were enriched in these peptides (Gupta et al., 2016), suggesting the importance of the PARKAR motif in peptide P1. A broad-spectrum cationic synthetic peptide, Peptide 1018, which inhibits biofilms by blocking the bacterial signaling molecule (p)ppGpp (de la Fuente-Núñez et al., 2014), was found to be effective in reducing multispecies oral biofilms (Wang et al., 2015).

Since biofilm formation is highly dependent on environmental conditions and on the surface properties such as charge and hydrophobicity of the material (Song et al., 2015), peptide P1 may be suitable for different formulations than existing anti-biofilm molecules. Conclusions from this work can inform future rational design of synthetic anti-biofilm peptides. In addition to future application as an anti-biofilm catheter coating against *S. aureus* infection (Heisig et al., 2014), it has potential to be used against oral biofilms on teeth or dental implant surfaces, alone or in combination with an antimicrobial such as chlorhexidine. The synthetic peptide P1 is a promising candidate for further development as an anti-biofilm agent against streptococci or other Gram-positive pathogens.

## Author Contributions

JMA designed the study, performed experiments, and wrote the manuscript. NMA provided assistance in designing and performing experiments, and contributed to data analysis and editing of the manuscript. JS-C, JM, and KM assisted with experiments, analyzed data, and contributed to writing the manuscript. EF provided critical feedback and support during the study.

## Conflict of Interest Statement

The authors declare that the research was conducted in the absence of any commercial or financial relationships that could be construed as a potential conflict of interest.

## References

[B1] Abraham N. M. Liu L. Jutras B. L. Yadav A. K. Narasimhan S. Gopalakrishnan V. (2017). Pathogen-mediated manipulation of arthropod microbiota to promote infection. *Proc. Natl. Acad. Sci. U.S.A.* 114 E781–E790. 10.1073/pnas.1613422114 28096373 PMC5293115

[B2] Basak S. Rajurkar M. Attal R. Mallick S. (2013). “Biofilms: a challenge to medical fraternity in infection control,” in *Infection Control* ed. Basak S. (Rijeka: InTech), 57–74. 10.5772/56704

[B3] Besingi R. N. Wenderska I. B. Senadheera D. B. Cvitkovitch D. G. Long J. R. Wen Z. T. (2017). Functional Amyloids in *Streptococcus mutans*, their use as targets of biofilm inhibition and initial characterization of SMU_63c. *Microbiology* 10.1099/mic.0.000443 [Epub ahead of print]. PMC5775903 28141493

[B4] Bjarnsholt T. Ciofu O. Molin S. Givskov M. Høiby N. (2013). Applying insights from biofilm biology to drug development–can a new approach be developed? *Nat. Rev. Drug Discov.* 12 791–808. 10.1038/nrd4000 24080700

[B5] Blanco L. P. Evans M. L. Smith D. R. Badtke M. P. Chapman M. R. (2012). Diversity, biogenesis and function of microbial amyloids. *Trends Microbiol.* 20 66–73. 10.1016/j.tim.2011.11.005 22197327 PMC3278576

[B6] Cardoso M. H. Ribeiro S. M. Nolasco D. O. de la Fuente-Núñez C. Felício M. R. Gonçalves S. (2016). A polyalanine peptide derived from polar fish with anti-infectious activities. *Sci. Rep.* 6:21385 10.1038/srep21385 PMC4768251 26916401

[B7] Cerf E. Sarroukh R. Tamamizu-Kato S. Breydo L. Derclaye S. Dufrêne Y. F. (2009). Antiparallel β-sheet: a signature structure of the oligomeric amyloid β-peptide. *Biochem. J.* 421 415–423. 10.1042/BJ20090379 19435461

[B8] Chen L. Ren Z. Zhou X. Zeng J. Zou J. Li Y. (2015). Inhibition of *Streptococcus mutans* biofilm formation, extracellular polysaccharide production, and virulence by an oxazole derivative. *Appl. Microbiol. Biotechnol.* 100 857–867. 10.1007/s00253-015-7092-1 26526453

[B9] Chen X. Hirt H. Li Y. Gorr S.-U. Aparicio C. Kreth J. (2014). Antimicrobial GL13K peptide coatings killed and ruptured the wall of *Streptococcus gordonii* and prevented formation and growth of biofilms. *PLoS ONE* 9:e111579 10.1371/journal.pone.0111579 PMC4221044 25372402

[B10] Das T. Sehar S. Koop L. Wong Y. K. Ahmed S. Siddiqui K. S. (2014). Influence of calcium in extracellular DNA mediated bacterial aggregation and biofilm formation. *PLoS ONE* 9:e91935 10.1371/journal.pone.0091935 PMC3961253 24651318

[B11] de la Fuente-Núñez C. Cardoso M. H. de Souza Cândido E. Franco O. L. Hancock R. E. W. (2016). Synthetic antibiofilm peptides. *Biochim. Biophys. Acta* 1858 1061–1069. 10.1016/j.bbamem.2015.12.015 26724202 PMC4809770

[B12] de la Fuente-Núñez C. Reffuveille F. Haney E. F. Straus S. K. Hancock R. E. W. (2014). Broad-spectrum anti-biofilm peptide that targets a cellular stress response. *PLoS Pathog.* 10:e1004152 10.1371/journal.ppat.1004152 PMC4031209 24852171

[B13] Di Luca M. Maccari G. Maisetta G. Batoni G. (2015). BaAMPs: the database of biofilm-active antimicrobial peptides. *Biofouling* 31 193–199. 10.1080/08927014.2015.1021340 25760404

[B14] Ding Y. Wang W. Fan M. Tong Z. Kuang R. Jiang W. (2014). Antimicrobial and anti-biofilm effect of Bac8c on major bacteria associated with dental caries and *Streptococcus mutans* biofilms. *Peptides* 52 61–67. 10.1016/j.peptides.2013.11.020 24309076

[B15] Donlan R. M. Costerton J. W. (2002). Biofilms: survival mechanisms of clinically relevant microorganisms. *Clin. Microbiol. Rev.* 15 167–193. 10.1128/CMR.15.2.167-193.2002 11932229 PMC118068

[B16] Frenkel E. S. Ribbeck K. (2015). Salivary mucins protect surfaces from colonization by cariogenic bacteria. *Appl. Environ. Microbiol.* 81 332–338. 10.1128/AEM.02573-14 25344244 PMC4272720

[B17] Gupta S. Sharma A. K. Jaiswal S. K. Sharma V. K. (2016). Prediction of biofilm inhibiting peptides: an in silico approach. *Front. Microbiol.* 7:949 10.3389/fmicb.2016.00949 PMC4909740 27379078

[B18] Haris P. I. Chapman D. (1995). The conformational analysis of peptides using fourier transform IR spectroscopy. *Biopolymers* 37 251–263. 10.1002/bip.360370404 7540054

[B19] Heisig M. Abraham N. M. Liu L. Neelakanta G. Mattessich S. Sultana H. (2014). Antivirulence properties of an antifreeze protein. *Cell Rep.* 9 417–424. 10.1016/j.celrep.2014.09.034 25373896 PMC4223805

[B20] Hirota K. Yumoto H. Miyamoto K. Yamamoto N. Murakami K. Hoshino Y. (2011). MPC-polymer reduces adherence and biofilm formation by oral bacteria. *J. Dent. Res.* 90 900–905. 10.1177/0022034511402996 21447697

[B21] Klein M. I. Hwang G. Santos P. H. S. Campanella O. H. Koo H. (2015). *Streptococcus mutans*-derived extracellular matrix in cariogenic oral biofilms. *Front. Cell. Infect. Microbiol.* 5:10 10.3389/fcimb.2015.00010 PMC4327733 25763359

[B22] Koo H. Falsetta M. L. Klein M. I. (2013). The exopolysaccharide matrix: a virulence determinant of cariogenic biofilm. *J. Dent. Res.* 92 1065–1073. 10.1177/0022034513504218 24045647 PMC3834652

[B23] Koo H. Xiao J. Klein M. I. Jeon J. G. (2010). Exopolysaccharides produced by *Streptococcus mutans* glucosyltransferases modulate the establishment of microcolonies within multispecies biofilms. *J. Bacteriol.* 192 3024–3032. 10.1128/JB.01649-09 20233920 PMC2901689

[B24] Lemos J. A. Abranches J. Koo H. Marquis R. E. Burne R. A. (2010). Protocols to study the physiology of oral biofilms. *Methods Mol. Biol.* 666 87–102. 10.1007/978-1-60761-820-1_7 20717780 PMC3130507

[B25] Lévesque C. M. Voronejskaia E. Cathy Y. Mair R. W. Ellen R. P. Dennis G. (2005). Involvement of sortase anchoring of cell wall proteins in biofilm formation by *Streptococcus mutans*. *Infect. Immun.* 73 3773–3777. 10.1128/IAI.73.6.3773 15908410 PMC1111851

[B26] Liao S. Klein M. I. Heim K. P. Fan Y. Bitoun J. P. Ahn S. J. (2014). *Streptococcus mutans* extracellular DNA is upregulated during growth in biofilms, actively released via membrane vesicles, and influenced by components of the protein secretion machinery. *J. Bacteriol.* 196 2355–2366. 10.1128/JB.01493-14 24748612 PMC4054167

[B27] Merritt J. H. Kadouri D. E. O’Toole G. A. (2011). Growing and analyzing static biofilms. *Curr. Protoc. Microbiol.* Chapter 1 Unit1B.1 10.1002/9780471729259.mc01b01s22 PMC4568995 18770545

[B28] Nagel L. Budke C. Dreyer A. Koop T. Sewald N. (2012). Antifreeze glycopeptide diastereomers. *Beilstein J. Org. Chem.* 8 1657–1667. 10.3762/bjoc.8.190 23209499 PMC3510999

[B29] Neelakanta G. Sultana H. Fish D. Anderson J. F. Fikrig E. (2010). Anaplasma phagocytophilum induces *Ixodes scapularis* ticks to express an antifreeze glycoprotein gene that enhances their survival in the cold. *J. Clin. Invest.* 120 3179–3190. 10.1172/JCI42868 20739755 PMC2929727

[B30] Oli M. W. Otoo H. N. Crowley P. J. Heim K. P. Nascimento M. M. Ramsook C. B. (2012). Functional amyloid formation by *Streptococcus mutans*. *Microbiology* 158 2903–2916. 10.1099/mic.0.060855-0 23082034 PMC4083658

[B31] Otsuka R. Imai S. Murata T. Nomura Y. Okamoto M. Tsumori H. (2015). Application of chimeric glucanase comprising mutanase and dextranase for prevention of dental biofilm formation. *Microbiol. Immunol.* 59 28–36. 10.1111/1348-0421.12214 25411090

[B32] Otter J. A. Vickery K. Walker J. T. Delancey Pulcini E. Stoodley P. Goldenberg S. D. (2015). Surface-attached cells, biofilms and biocide susceptibility: implications for hospital cleaning and disinfection. *J. Hosp. Infect.* 89 16–27. 10.1016/j.jhin.2014.09.008 25447198

[B33] Otto M. (2014). Physical stress and bacterial colonization. *FEMS Microbiol. Rev.* 38 1250–1270. 10.1111/1574-6976.12088 25212723 PMC4227950

[B34] Pletzer D. Hancock R. E. W. (2016). Antibiofilm peptides: potential as broad-spectrum agents. *J. Bacteriol.* 198 2572–2578. 10.1128/JB.00017-16.Editor 27068589 PMC5019066

[B35] Rendueles O. Kaplan J. B. Ghigo J. M. (2013). Antibiofilm polysaccharides. *Environ. Microbiol.* 15 334–346. 10.1111/j.1462-2920.2012.02810.x 22730907 PMC3502681

[B36] Schneider C. A. Rasband W. S. Eliceiri K. W. (2012). NIH Image to ImageJ: 25 years of image analysis. *Nat. Methods* 9 671–675. 10.1038/nmeth.2089 22930834 PMC5554542

[B37] Song F. Koo H. Ren D. (2015). Effects of material properties on bacterial adhesion and biofilm formation. *J. Dent. Res.* 94 1027–1034. 10.1177/0022034515587690 26001706

[B38] Taglialegna A. Lasa I. Valle J. (2016a). Amyloid structures as biofilm matrix scaffolds. *J. Bacteriol.* 198 2579–2588. 10.1128/JB.00122-16 27185827 PMC5019065

[B39] Taglialegna A. Navarro S. Ventura S. Garnett J. A. Matthews S. Penades J. R. (2016b). Staphylococcal bap proteins build amyloid scaffold biofilm matrices in response to environmental signals. *PLOS Pathog.* 12:e1005711 10.1371/journal.ppat.1005711 PMC4915627 27327765

[B40] Thi P. Nguyen M. Falsetta M. L. Hwang G. Gonzalez-Begne M. Koo H. (2014). a-Mangostin Disrupts the Development of *Streptococcus mutans* biofilms and facilitates its mechanical removal. *PLoS ONE* 9:e111312 10.1371/journal.pone.0111312 PMC4211880 25350668

[B41] Torrent M. Valle J. Nogués M. V. Boix E. Andreu D. (2011). The generation of antimicrobial peptide activity: a trade-off between charge and aggregation? *Angew. Chem. Int. Ed.* 50 10686–10689. 10.1002/anie.201103589 21928454

[B42] Wang Z. de la Fuente-Núñez C. Shen Y. Haapasalo M. Hancock R. E. W. (2015). Treatment of Oral Multispecies Biofilms by an Anti-Biofilm Peptide. *PLoS ONE* 10:e0132512 10.1371/journal.pone.0132512 PMC4500547 26168273

[B43] Xiao J. Klein M. I. Falsetta M. L. Lu B. Delahunty C. M. Yates J. R. (2012). The exopolysaccharide matrix modulates the interaction between 3D architecture and virulence of a mixed-species oral biofilm. *PLoS Pathog.* 8:e1002623 10.1371/journal.ppat.1002623 PMC3320608 22496649

[B44] Xiao J. Koo H. (2010). Structural organization and dynamics of exopolysaccharide matrix and microcolonies formation by *Streptococcus mutans* in biofilms. *J. Appl. Microbiol.* 108 2103–2113. 10.1111/j.1365-2672.2009.04616.x 19941630

