# Supplementary figures and images for: Anti-Biofilm Activity of a Self-Aggregating Peptide against Streptococcus mutans
Source: Front Microbiol. 2017 Mar 24;8:488. doi: 10.3389/fmicb.2017.00488 (PMC5364132; doi:10.3389/fmicb.2017.00488)

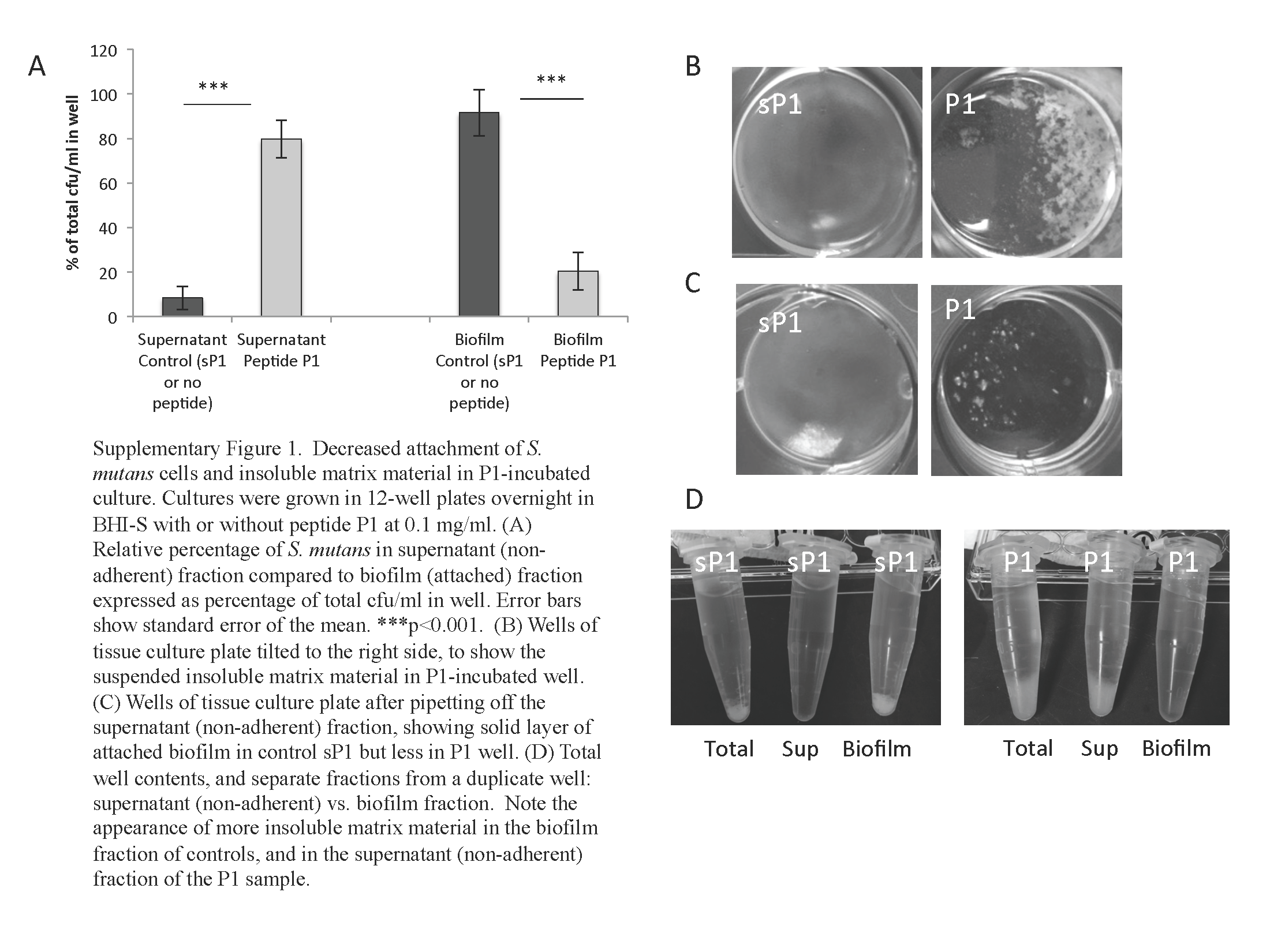

Supplement: Supplementary file 1 [file Image_1.TIFF]
